# Inflammatory and intestinal permeability biomarkers in healthy participants on long term vegan, vegetarian, omnivore and low-carbohydrate high-fat diet

**DOI:** 10.1038/s41598-023-44233-0

**Published:** 2023-10-12

**Authors:** Zala Jenko Pražnikar, Karin Šik Novak, Nives Bogataj Jontez, Ana Petelin, Nina Mohorko, Saša Kenig

**Affiliations:** University of Primorska, Faculty of Health Sciences, Polje 42, 6310 Izola, Slovenia

**Keywords:** Biomarkers, Health care, Risk factors

## Abstract

Vegan, vegetarian and low-carbohydrate high fat (LCHF) diets can all offer several health benefits, if food choices are appropriate. In most studies examining their effects on systemic inflammation, participants were either overweight, on a weight loss programme or not matched for BMI, or had a pre-existing condition such as type 2 diabetes mellitus or hypertension. Little is known about the effects of dietary patterns on healthy and normal weight individuals. The aim of the present study was therefore to assess and directly compare inflammatory and intestinal permeability status in healthy participants following aforementioned or omnivore diet for at least 6 months. In this cross-sectional study, we measured the inflammatory biomarkers IL-6, TNF-α and CRP, and the markers of intestinal permeability LBP and zonulin, along with the analysis of lifestyle aspects, dietary intakes and physical activity, in 89 healthy participants. The groups were matched for sex, age and BMI. There were no differences in any of the measured parameters between the four groups and we found no strong correlations with dietary intakes. Using cluster analysis, participants were divided into eight clusters with more or less favourable inflammatory profiles; all clusters contained representatives of all patterns and all patterns were represented in each cluster. Significant differences between clusters were in the intake of mono-unsaturated fatty acids, ω-3/ω-6 ratio, phase angle and working two shifts. In healthy, normal-weight individuals, inflammatory status therefore does not depend on the dietary pattern itself, but is rather more complexly regulated and associated with dietary and non-dietary factors.

## Introduction

In many Western countries, there is a growing trend towards different types of dietary patterns such as vegetarian, vegan and low carbohydrate high fat (LCHF)^1^. Vegetarian and vegan diets are typically rich in all types of fruits, vegetables, whole grains, legumes, nuts and various soy products^2^, while LCHF diets include high amounts of fats, meat, poultry, fish, eggs and cheese, red and processed meats^3^, as well as olive oil, avocados and green vegetables^4^. In recent years, there has been a growing trend towards veganism in particular, due to a growing awareness of the environmental problems associated with livestock farming and compassion for animals. However, more and more people are turning to a special diet because of the potential health benefits^5^. In fact, there is scientific evidence that vegan and vegetarian diet, as well as a LCHF diet, can influence metabolism in a way that it is protective against many chronic diseases such as obesity, type 2 diabetes mellitus (T2DM), cardiovascular diseases, or cancer^3,6^.

Inflammatory biomarkers may act as intermediate risk factors in the development of chronic diseases. Elevated levels of inflammatory markers such as high-sensitivity C-reactive protein (CRP), or tumor necrosis factor alpha (TNF-α) have been associated with pathogenetic mechanisms of obesity, T2DM and cardiovascular diseases^7^. CRP is predominantly synthesized and secreted by hepatocytes in response to proinflammatory cytokines such as TNF-α, interleukin-1 (IL-1) and interleukin-6 (IL-6). In a meta-analysis of 23 prospective studies, high CRP level showed a combined risk ratio of 1.60 for coronary heart disease compared to low CRP levels^8^.

Low-grade chronic inflammation is also linked to gut dysbiosis and disrupted intestinal barrier. Higher levels of zonulin, a ~ 47-kDa protein, that reversibly regulates intestinal permeability by modulating intercellular tight junctions^9^ and has been proposed as a marker of intestinal permeability^10^, have been associated with higher waist circumference, diastolic blood pressure, and fasting glucose, as well as increased risk of metabolic diseases^11^. This suggests that bacterial endotoxins may play an important role in the development of the metabolic and vascular abnormalities common in obesity and diabetes-related diseases. Moreover, impaired fasting blood glucose is associated with elevated serum lipopolysaccharide and zonulin levels, which are independent and unrelated markers of increased intestinal permeability^12^.

Understanding the role of diet as a modifiable determinant of these pathophysiological pathways may therefore hold the key to inflammation-related disease prevention. Recent evidence proposed that inflammatory biomarker profiles can be modulated by plant-based diets rich in anti-inflammatory nutrients, showing an attenuation of inflammatory markers compared to omnivore diets^13,14^. In addition, total energy intake tends to be lower on vegetarian and vegan diets, which may also contribute to the reduction of inflammation. It is worth noting that there may be individual differences, and some studies have found little or no difference in CRP levels between vegetarian, vegan and omnivore groups^15^. As for the LCHF diet, some studies have suggested that it may also be associated with lower levels of inflammatory markers, such as CRP and IL-6, compared to high-carbohydrate diets. However, it is possible that the observed reduced inflammation is due to the weight loss rather than the change in dietary pattern itself, considering that LCHF diet is often promoted as a weight loss strategy. Knowledge about the long-term effects of the LCHF diet on inflammation is limited.

While some studies have examined the associations between vegetarian diet and inflammatory biomarkers, there is still a lack of comprehensive scientific research on the relationship between an exclusively vegan or LCHF diet and inflammatory biomarkers. This is especially true for cases in which the dietary patterns are used in long-term and are self-planned. The aim of the present study is therefore to assess the inflammatory and intestinal permeability status in participants who followed a vegan, vegetarian, LCHF or omnivore diet for at least six months without the supervision of a nutrition expert. Since age and BMI are known to correlate with inflammation^16,17^, participants in the dietary groups were matched for BMI, sex and age.

## Results

### General characteristics of study subjects

The general characteristics of the 89 participants on either vegan (n = 24), vegetarian (n = 21), LCHF (n = 20), or omnivore diet (n = 24) are shown in Table 1. Due to the selection of homogeneous groups based on sex, age and BMI, there were no significant differences in these parameters (in all groups, approximately 1/3 of participants were male, mean BMI ranged from 21.7 to 23.3 kg/m^2^, and mean age ranged from 33.6 years in the vegan group to 39.4 years in the LCHF group). In addition, we found no differences in waist circumference, fat mass, energy intake, physical activity, smoking status, educational status, socioeconomic status, or alcohol consumption (all p > 0.05). However, as expected, the four dietary groups differed significantly in protein, carbohydrate, and fat intake. Total and animal protein intake was the highest in LCHF and the lowest in vegan group, while it was the opposite for plant protein, carbohydrates, and fibre. Only vegan and vegetarian groups achieved the recommended daily intake^18^ of dietary fibre (> 30 g).

**Table 1:** General characteristics of study participants (n = 89).

	Omnivore (n = 24)	Vegan (n = 24)	Vegetarian (n = 21)	LCHF (n = 20)	p-value
Sex (%M/%F)	33.3/66.7	33.3/66.7	33.3/66.7	30.0/70.0	0.99
Age (years)	36.2 (10.4)	33.6 (9.6)	37.1 (10.8)	39.4 (6.9)	0.10
BMI (kg/m^2^)	22.2 (3.0)	21.7 (2.2)	22.3 (2.4)	23.3 (3.2)	0.38
WC (cm)	75.5 (9.8)	74.5 (7.5)	76.2 (8.8)	76.8 (8.3)	0.85
Fat mass (%)	21.8 (7.3)	19.7 (8.2)	22.0 (7.2)	21.5 (7.1)	0.71
Phase angle (°)	6.6 (1.0)	6.4 (0.9)	6.2 (0.8)	6.5 (1.0)	0.56
Not working (%)	4.2	12.5	19.0	10.0	0.23
One-shift work (%)	50.0	41.7	52.4	75.0	
Two-shifts work (%)	37.5	25.0	14.3	10.0	
Flexible work (%)	8.3	20.8	14.3	5.0	
PA (MET/day)	11.7 (9.3)	11.8 (11.4)	9.7 (8.7)	7.5 (6.3)	0.44
Smoking (%)	25	12	4.8	20	0.27
Energy intake (kcal)	2162 (800)	2141 (716)	2143 (664)	1981 (568)	0.90
Total protein (%EI)	16.0 (3.1)	12.3 (2.8)	13.1 (3.7)	22.5 (5.7)	** < 0.01**^**a,c,d,e**^
Total CHO (%EI)	46.6 (7.1)	59.0 (11.2)	50.5 (10.4)	9.4 (6.0)	** < 0.01**^**a,c,d,e**^
Total fats (%EI)	35.5 (7.1)	27.6 (9.8)	36.1 (9.3)	66.2 (8.2)	** < 0.01**^**a,c,d,e,f**^
SFA (%EI)	10.7 (2.7)	5.8 (3.2)	9.8 (3.5)	25.1 (6.1)	** < 0.01**^**a,c,d,e,f**^
MUFA (%EI)	10.2 (3.8)	9.6 (4.7)	10.9 (5.1)	22.0 (6.7)	** < 0.01**^**c,d,e**^
ω-3 PUFA (%EI)	0.6 (0.6)	0.7 (0.7)	0.7 (0.7)	1.1 (0.7)	** < 0.01**^**c,d**^
ω-3/ω-6 PUFA (ratio)	0.2 (0.2)	0.4 (0.7)	0.2 (0.2)	0.3 (0.2)	0.33
Dietary fiber (g)	27.6 (17.6)	55.4 (48.1)	35.1 (13.9)	16.3 (22.9)	** < 0.01**^**a,c,d,e**^
Alcohol (units/week)	2.1 (3.8)	1.0 (1.3)	1.3 (1.8)	2.0 (3.6)	0.93

Discrete variables are expressed as frequency (%). All other values are expressed as mean with standard deviation in brackets. Groups were compared with ANOVA or Kruskal–Wallis test and statistically significant differences determined with a post-hoc test are marked as follows: ^a^Omnivore-Vegan, ^b^Omnivore-Vegetarian, ^c^Omnivore-LCHF, ^d^Vegan-LCHF, ^e^Vegetarian-LCHF, ^f^Vegan-Vegetarian; BMI: body mass index, CHO: carbohydrate; EI: daily energy intake; LCHF: low-carbohydrate high-fat; MUFA: monounsaturated fatty acids; PA: physical activity; PUFA: polyunsaturated fatty acids; SFA: saturated fatty acids; WC: waist circumference.

Significant values are in bold.

### Inflammatory and intestinal permeability biomarkers

As shown in Table 2, we observed no significant differences in inflammatory biomarkers, i.e. CRP, IL-6, TNF-α, and intestinal permeability biomarkers zonulin and lipopolysaccharide binding protein (LBP) between four dietary groups (all p > 0.05). When subjects were divided into two groups according to the duration of adherence to a particular diet (less than 3 years and > 3 years), we found that subjects who had been on a vegan diet for more than 3 years had statistically significant lower zonulin levels compared to those on omnivore diet [1.22 ± 0.85 (vegan) vs. 1.94 ± 1.22 (omnivore); t = − 2.03, p = 0.05]. In the vegan group, serum zonulin levels even correlated significantly with the duration of the vegan diet (r = − 0.561, p = 0.015). Such significant correlations were not found in other groups.

**Table 2:** Inflammatory and intestinal permeability biomarkers in the four dietary groups (n = 89).

		Omnivore (n = 24)	Vegan (n = 24)	Vegetarian (n = 21)	LCHF (n = 20)	p-value^a^	p-value^b^
CRP [mg/l]		0.37 (2.01)	0.34 (0.66)	0.31 (0.80)	0.49 (0.64)	0.63	0.71
IL-6 [pg/ml]		1.30 (2.80)	1.01 (1.41)	1.22 (0.59)	1.16 (3.98)	0.94	0.97
TNF-α [pg/ml]		0.37 (0.27)	0.52 (2.99)	0.57 (0.64)	0.67 (1.93)	0.15	0.15
Zonulin [ng/ml]		1.67 (1.20)	1.30 (0.75)	1.86 (10.96)	1.26 (0.92)	0.36	0.45
LBP [µg/ml]		3.97 (1.20)	3.56 (1.60)	3.92 (1.59)	4.25 (1.95)	0.61	0.68

The values are expressed as median with IQR in brackets; ^a^Kruskal-Wallis test; ^b^Model (nonparametric ANCOVA) adjusted for age, sex, smoking status, education, waist circumference, physical activity, alcohol consumption. CRP: C-reactive protein; IL: interleukin; LCHF: low-carbohydrate high-fat; LBP: lipopolysaccharide binding protein; TNF-α: tumour necrosis factor α.

### Correlation analysis between inflammatory and intestinal permeability biomarkers and nutritional factors

**Figure 1:**
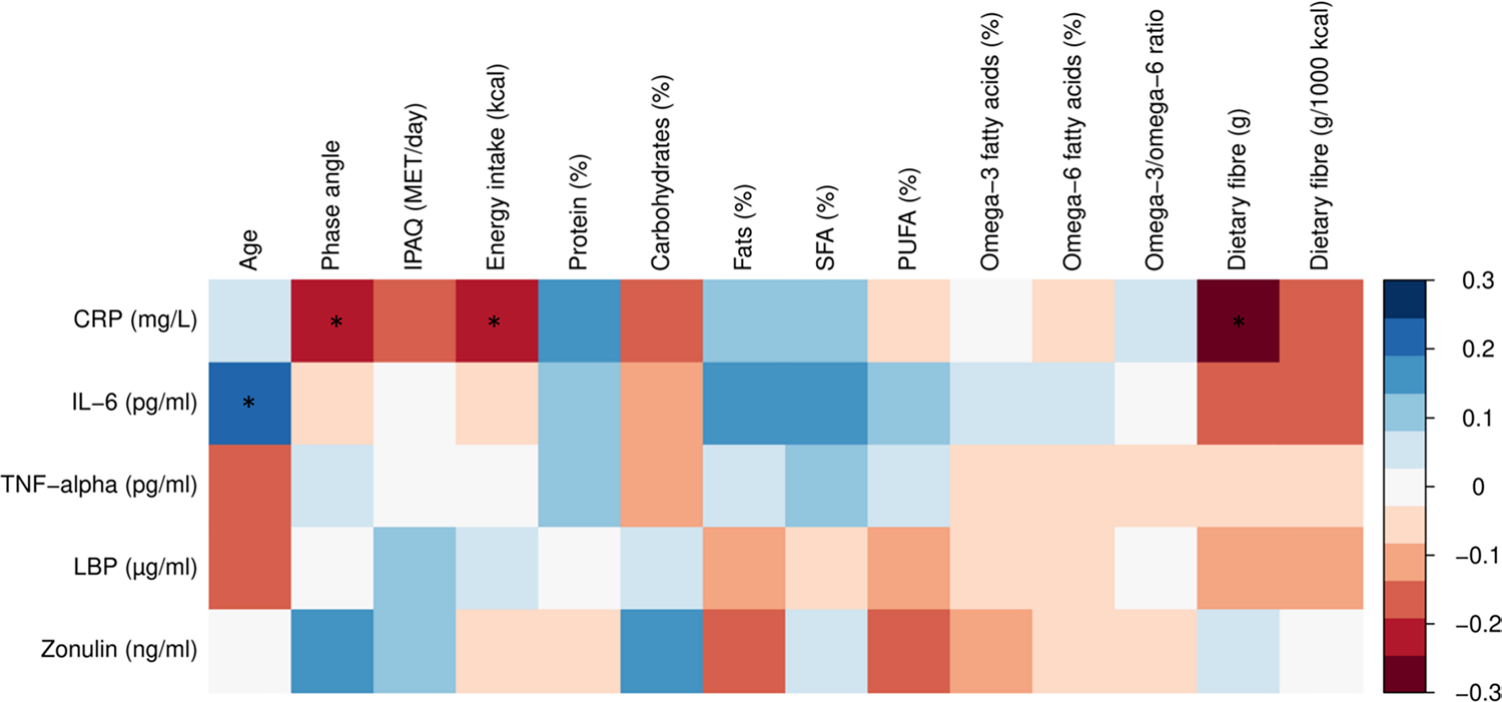
Heatmap representing Spearman correlations of inflammatory biomarkers and biomarkers of intestinal permeability with age, phase angle, IPAQ and nutritional factors (n = 89). *Statistically significant associations, red—negative correlation, blue—positive correlation. CRP: C-reactive protein; IL: interleukin; IPAQ: International Physical Activity Questionnaire; LBP: lipopolysaccharide binding protein; TNF-α: tumour necrosis factor α; MET: metabolic equivalent of task; PUFA: polyunsaturated fatty acids; SFA: saturated fatty acids.

To investigate the relationship between inflammatory and intestinal permeability biomarkers and nutritional factors or parameters of nutritional status and anthropometric parameters, correlation analysis was performed. In our study, there were significant negative associations between CRP and phase angle, energy intake and intake of dietary fibre (Fig. 1). IL-6 was significantly positively associated with age. All four associations were weak (Fig. 1). For zonulin, TNF-α and LBP there was no association with any of the examined parameters. In sensitivity analyses, exclusion of participants taking antiallergic drugs or analgesics, exclusion of extreme levels of inflammatory biomarkers, or additional adjustment for diet type did not significantly alter the observed associations (data not shown).

### Hierarchical cluster analysis based on biomarkers of inflammation and intestinal permeability

**Figure 2:**
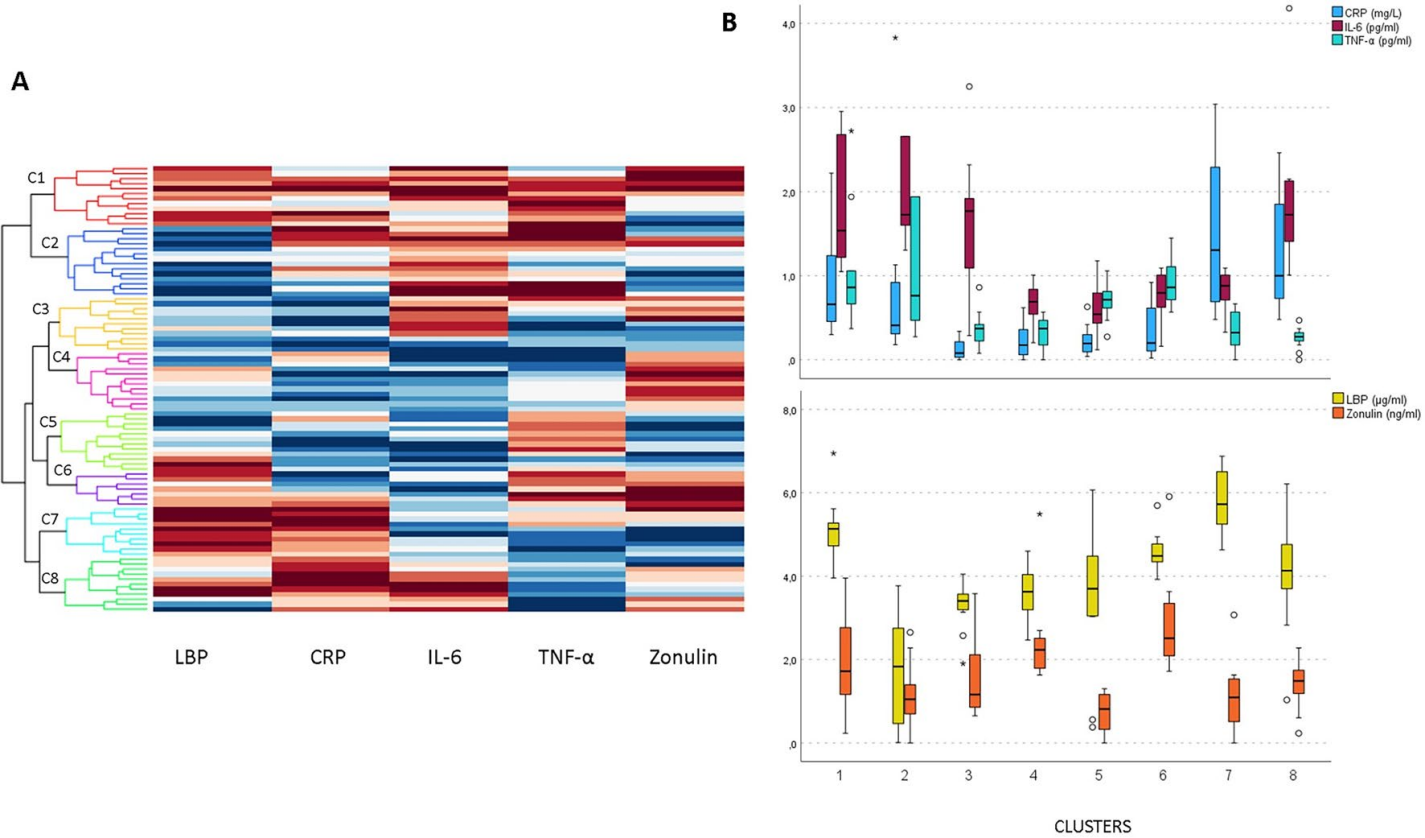
Hierarchical cluster analysis according to CRP, IL-6, TNF-α, LBP, and zonulin. (**A**) Participants are distributed into eight clusters, named C1 to C8. The deeper the red colour, the higher the level of the marker; the deeper the blue colour, the lower the level, white colour represents a median value. (**B**) Serum levels of inflammatory and intestinal permeability biomarkers in each cluster. Medians and 95th percentiles are presented in box plots. CRP: C-reactive protein; IL: interleukin; LBP: lipopolysaccharide binding protein; TNF-α: tumour necrosis factor α. Outliers are labelled and shown as star in the case of male participant and as circle in the case of the female participant.

Because no differences in biomarkers of inflammation or intestinal permeability were found between dietary patterns and no strong associations were found between nutritional factors and biomarkers, we performed a hierarchical cluster analysis according to CRP, IL-6, TNF-α, LBP, and zonulin. In Fig. 2, higher values are coloured in red (worse inflammatory profile), whereas the blue colour represents lower values of each biomarker. When all participants were classified according to their inflammatory profile, eight different clusters were observed (Fig. 2A).

None of the clusters were specific to a particular type of diet (Table 3). In addition, all four dietary groups were present in each cluster, suggesting that the inflammatory profile was not related to dietary pattern in BMI-, age- and sex-matched groups of healthy participants. The highest proportion of participants on omnivore diet was found in C8, followed by C3, C4 and C5. Participants adhering to vegan diet were found in all clusters, with the highest proportion in C3, C7 and then C2. Higher proportions of vegetarians were found in C2 and C6, whereas those adhering to LCHF diet were mainly found in C1 and C2.

**Table 3:**
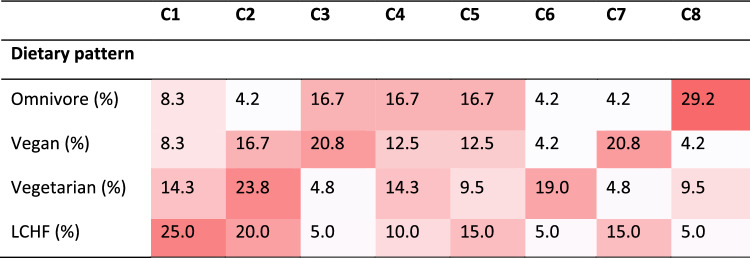
Classification of participants on different diets to eight clusters.

For each dietary group, the distribution of participants to the eight clusters is presented. The deeper the red color, the higher the proportion of participants in one cluster. C: cluster; LCHF: low-carbohydrate high-fat.

Cluster C1 had the worst inflammatory profile with all five biomarkers above the median values (Fig. 2B) and particularly high TNF-α and LBP. In this cluster, 42% of participants were following a LCHF diet, 25% vegetarian, and less were following vegan and omnivore diet. Similar phenotype (except for LBP) was observed also in cluster C2, in which 36% were on vegetarian, 28% on vegan, 28% on LCHF, and 7% on omnivore diet (Fig. 2, Table 4). Cluster C3 had significantly better inflammatory profile, especially in terms of CRP and TNF-α levels, which were significantly lower compared with the other clusters, particularly clusters 1, 2, 6, and 7; in this cluster 46% were adhering to vegan and 36% to omnivore diet (Fig. 2, Table 4). The inflammatory profile was the most favourable in clusters C4 and C5; since in C4, only zonulin, whereas in C5, only TNF-α, were above the median value; in both clusters all four dietary patterns were almost evenly distributed. In cluster C6, the highest levels of TNF-α and zonulin were observed and LBP was also high; in contrast, CRP and IL-6 were average (Fig. 2). C6 was represented mostly by those on vegetarian diet (57%) (Table 4). The highest CRP and LBP levels were observed in cluster C7 (Fig. 2). In this cluster, 50% were adhering to vegan and 30% to LCHF diet (Table 4). Cluster C8, with low TNF-α and zonulin, but elevated CRP, LBP, and IL-6, consisted mostly of omnivores (64%, Table 4).

**Table 4:**
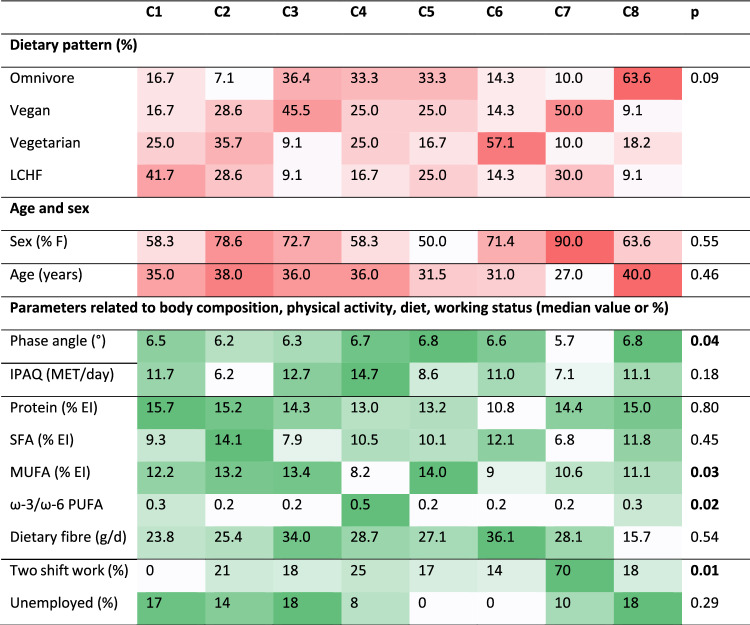
Characteristics of specific clusters.

The characteristics of participants that compose each cluster are presented. The color intensity increases with increasing value. C: cluster; EI: daily energy intake; IPAQ: International Physical Activity Questionnaire; LCHF: low-carbohydrate high-fat; MET: metabolic equivalent of task; MUFA: monounsaturated fatty acids; PUFA: polyunsaturated fatty acids; SFA: saturated fatty acids.

Significant values are in bold.

### Differences between participants classified in specific clusters

Since the participants adhering to each dietary pattern were found in all clusters, we have looked for other specifics that participants in each cluster may have in common (Table 4). We compared the clusters with favourable (C3, C4, C5) or less favourable inflammatory profile (C1, C2, C7). Compared with the clusters with less favourable inflammatory profiles, participants in cluster C4 had better ratios of ω-3/ω-6 fatty acid intake (Table 4). Additionally, participants in C4 were more physically active and had higher phase angle. Also, participants in C3 tended to be more physically active, consumed higher amounts of fibre and less SFA compared to clusters C1 and C2. The main difference between participants in C4 and C5 was the intake of MUFA and PUFA; participants in C4 had a better ratio of ω-3/ω-6 fatty acids and were more physically active, while those in C5 had the highest intake of MUFA. In contrast, in cluster C7, where the highest CRP and LBP levels were observed, the participants had low ω-3/ω-6 ratio, the lowest phase angle, lower IPAQ, the participants were the youngest and there was the highest proportion of females. Another difference between the participants in C7 and the others was in the working schedule—almost 70% of the participants in C7 worked two shifts, which in our case was related to a worse inflammatory profile. In C6, in which the participants adhering to vegetarian diet predominate, we found the highest intake of fibre. Participants in group C8 on the other hand had the highest phase angle, they were very active but also somewhat older than those in other clusters. ANOVA comparing all clusters revealed significant differences in phase angle, intake of MUFA, ω-3/ω-6 ratio and working schedule (p < 0.05; Table 4), while other differences were only significant when individual groups were compared.

## Discussion

Systemic low-grade inflammation is linked to various diseases^19^ and there is a growing body of studies suggesting that different dietary patterns can have varying effects on inflammation markers^20–22^. While the specific mechanisms underlying these effects are not fully understood, it is believed that dietary factors may influence inflammation through several pathways, including oxidative stress, gut microbiota, and immune function.

In the present cross-sectional study, we investigated inflammatory biomarkers in BMI-, sex-, and age-matched groups of participants exclusively adhering to either vegan, vegetarian, LCHF or omnivore diet. Overall, we observed no significant differences in CRP, TNF-α and IL-6, and there were no differences in the biomarkers of intestinal permeability zonulin and LBP among the four groups. We observed no strong associations between dietary intakes and the investigated parameters. In addition, cluster analysis showed that none of the clusters was specific for a particular type of diet and that all four dietary groups were represented in each cluster. Overall, this analysis showed that dietary pattern had no significant effect on the inflammatory profile and markers of intestinal permeability in normal-weight, BMI-, age- and sex-matched healthy adults. Interestingly, in a similar comparison, we have found no major differences in glucose and triglyceride levels, whereas cholesterol levels were somewhat higher in LCHF group^23^. Here, participants in the clusters significantly differed in the intake of MUFA, ω-3/ω-6 ratio, phase angle, and work schedule.

Previously, some studies have already investigated the association of vegan or vegetarian diet with inflammation, while there is little information on LCHF diets. This is particularly true for healthy individuals who are not overweight, are not on a weight loss program or do not have pronounced risk factors for cardiovascular diseases. Menzel et al.^24^ and Sebekova et al.^25^ found no differences in CRP levels between vegan and omnivore group. The second study focused only on CRP as the inflammatory marker, while Menzel et al.^24^ conducted a comprehensive investigation of a broad range of inflammatory biomarkers in exclusive vegan compared with omnivore groups and, in agreement with our results, found no differences. In contrast, studies on participants with higher BMI, fat mass, and waist circumference, who were classified as overweight observed higher CRP levels in omnivore compared to vegan participants^26^.

Compared to vegan, vegetarian dietary pattern in relation to inflammatory status has been investigated in more studies. Craddock et al.^27^ in a systematic review and meta-analysis report that CRP decreased in 9/19 observational studies and in 4/7 interventional studies, whereas it remained unchanged in other studies. It should be pointed out that in these studies, participants were not matched for BMI^28^ and that in interventions they either had T2DM, rheumatoid arthritis or were obese. In addition, the authors noted that apart from CRP other inflammatory and immune biomarkers of interest besides CRP were not examined or were examined only in single studies, thus limiting the conclusions^27^. However, Lee et al.^29^ showed that individuals with a higher "vegetable pattern" levels had lower CRP concentrations as well as higher antioxidant intake. Again, the association was more pronounced in male participants with hypertension. Interestingly, Haghighatdoost et al.^30^ emphasized a trend towards lower CRP concentrations in participants who had been on a vegetarian diet for at least 2 years, whereas no significant effect was observed in participants who had been on a vegetarian diet for at least 6 months but less than 2 years. Consistent with these observations, Menzel et al.^15^ found that 'long-term' vegan group (> 4.8 years) tended to have lower hsCRP levels compared to those who were on vegan diet ≤ 4.8 years. We found similar result only for the vegan group—the "long-term" vegan group (> 3 years) tended to have lower zonulin levels than omnivores, and the level of zonulin negatively correlated with the duration of adherence to the vegan diet. No such correlation was observed for CRP and other markers in our study.

In recent years, LCHF diets have become popular for the treatment of obesity and T2DM^31^, since they do not cause postprandial hyperglycemic spikes. These are associated with the formation of reactive oxygen species (ROS), which disrupt insulin signalling, enhance the progression of insulin resistance^32,33^ and affect blood levels of some inflammatory biomarkers. In fact, they promote the expression of pro-inflammatory molecules such as MCP-1, TNF-α, IL-6, IL-1β, along with AP-1, as well as FOXO more than chronic hyperglycaemia^34,35^. However, little is known about the effects of a LCHF diet on inflammation in healthy individuals. The present results suggest that such a diet is not associated with an increase or decrease in circulating inflammatory and intestinal permeability biomarkers in healthy adults compared to omnivore, vegan and vegetarian diets. Previous studies are inconclusive and generally do not focus on healthy normal-weight individuals. For example, a strict low-carbohydrate diet was associated with an increase in circulating levels of CRP, but not the inflammatory cytokine IL-6, in apparently healthy but overweight participants^36^, whereas in individuals with T2DM, a 2-year LCHF diet resulted in a significant decrease in CRP levels compared with participants following a high-carbohydrate, low-fat diet^37^. In participants with metabolic syndrome or obesity, a short-term ketogenic diet also resulted in positive cardiometabolic effects^38–40^. In T2DM patients, a one-year dietary intervention with ketosis also resulted in lower cardiovascular risk^41^, and in obese and overweight adolescents and adults, a diet based on low glycemic index foods also improved inflammatory, metabolic, as well as cardiovascular risk factors^42^.

The selection of a dietary pattern itself is therefore not the determining factor for the inflammatory status. This is not surprising as any diet may or may not consist of healthy foods that provide an optimal supply of nutrients. For example, a person following an LCHF diet may choose predominantly plant or animal fat sources, just as both fried potatoes or diverse vegetables and grain products would be considered vegan, but would provide a very different profile of nutrients. It is known that a number of nutrients, as well as non-nutritive bioactive components, and other lifestyle factors, can influence different health parameters^43^. We have, for example, shown previously, that the intake of SFA was the strongest predictor for increased cholesterol found in participants following LCHF diet^23^. Regarding inflammation, long-chain ω-3 fatty acids, particularly eicosapentaenoic acid and docosahexaenoic acid, can modulate the expression of inflammation-related genes^22^ and more importantly, they are precursors of molecules such as resolvins, maresins, and protectins involved in the resolution of inflammation^44^. We show here that the ratio of ω-3/ω-6 was the highest in participants in cluster C4 that was characterized by the most beneficial inflammatory profile. Consistent with our findings, studies using transgenic mouse models have shown that the ratio of ω-3/ω-6 acids in tissues has a critical impact on chronic diseases, including cancer and inflammatory diseases, and also influences the microbiome and the therapeutic effect of a high ω-3/ω-6 acids on a number of diseases has been demonstrated^45^. Of the dietary factors, intakes of MUFA, PUFA, SFA and dietary fiber are also relevant—all these parameters are known to be associated with inflammation^46^. However, regardless of the fact, that our participants on LCHF diet had the lowest intake of fiber and the highest intake of SFA, they can still be found in all different clusters, suggesting more complex regulation.

It is thus obvious, that other than dietary factors contribute to the inflammatory status. Indeed, our results suggest that phase angle differs between clusters with different inflammatory and intestinal permeability profiles. This factor may depend on the diet but also physical activity^47^. In fact, low physical activity has been found to be directly related to higher levels of CRP and proinflammatory cytokines in healthy individuals^48^. It should be pointed out that IL-6 can be derived from muscles during physical activity and under such conditions due to the combination with other myokines does not act as a proinflammatory cytokine^49^. An important difference between clusters in our study, night shift work, was also found to increase the risk for metabolic syndrome and is suspected to be among causal factors for obesity, T2DM, and cardiovascular disease^50^. The increasing exposure to artificial light, particularly the blue spectrum, which increases nocturnal wakefulness leading to disruption of circadian rhythms^51^ promotes inflammation^52^. In addition, rotating shift workers were shown to have irregular and more frequent meals, increased snacking and total 24-h energy intake and overall less appropriate choice of foods^53^. The contribution of other factors can not be excluded, especially since we have recently shown that the gut microbiota composition could be predicted with a 91% accuracy based on 26 factors, including anthropometric measures, serum biomarkers, lifestyle factors, gastrointestinal symptoms, psychological factors, and specific nutrients intake^54^. Since intestinal permeability and consequently the inflammatory status are both linked to the microbiota composition, the same factors could be considered relevant.

The main advantage of the present study in comparison with previous research is the fact that we directly compared four dietary groups, while pre-existing studies mostly compared only one separate pattern to omnivore or more often Western diet. The comprehensive data as a result of the standardized procedures, including the collection of blood, in combination with extensive information from questionnaires, dietary assessment by 3-day weighting protocol and anthropometric measurements, enabled us to adjust for the most important potential confounders. However, some limitations of our study deserve to be mentioned. The sample of 89 participants is relatively small and the cross-sectional design does not allow for causal inferences. The study included middle-aged healthy Slovenian men and women, and therefore the results may not be generalizable to other populations, such as other ethnicities or age groups. Participants were given detailed instructions on how to correctly report food intake, but, although food diaries and FFQs are standardly used, they are based on self-report, which is less reliable than direct monitoring and may therefore be considered a limitation.

## Conclusion

Overall, the main conclusion of the present study is that dietary pattern itself has no significant influence on inflammation and intestinal permeability in healthy, normal-weight participants. Regardless of dietary pattern, participants in vegan, vegetarian, omnivore or LCHF group may or may not have a favourable inflammatory and intestinal permeability profile, which is linked to dietary factors such as ω-3/ω-6 ratio and MUFA intake, but also other factors such as phase angle and work schedule. It should be noted that our participants were normal weight, under 60 years of age and healthy, and they had an above-average interest in nutrition and although they did not have guidance from a dietitian, their healthy eating index was relatively high^23^. Considering all these facts, the observed overall low levels of inflammatory markers are not surprising. However, since we reported before that none of the groups fully met the recommendations for macro- and micro-nutrient intakes, the choice of healthy foods should be suggested to the participant of all dietary patterns, regardless of the fact that their inflammatory status was not (yet) majorly affected.

## Methods

### Study design and participants

The present study named »The Link between Diets and Health Indicators (DIETE)« is a cross-sectional study. The study protocol was approved by the Slovenian National Medical Ethics Committee (No. 0120-557/2017/4 and 53/03/15) and was registered on ClinicalTrials.gov (Identifier: NCT04347213). All procedures were performed in agreement with the Declaration of Helsinki and informed consents were signed by all the participants. The recruitment and measurements were performed between February 2020 to October 2021 at the Faculty of Health Sciences, University of Primorska, Izola, Slovenia.

Detailed protocol for the selection of participants is published previously^54^. Briefly, participants adhering to four distinct dietary patterns, namely omnivore, vegan, vegetarian and LCHF diet, were recruited through newspapers and on social media. Asymptomatic adults with an unchanged eating pattern for at least six months prior to participation in the study and the following criteria were recruited: age between 20 and 60 years, BMI between 18.5 and 30 kg/m^2^, not taking any medications or antibiotics three months prior to participation, not pregnant or breastfeeding, no significant change in body mass three months prior to participation. The criteria for the inclusion into one of the groups were: complete exclusion of meat, meat products and fish for the vegetarian group and exclusion of all animal products for the vegan group. For the LCHF group participants had to have the intake of carbohydrates below 26%EI and the intake of fat above 50%EI. The 3 day-dietary diaries and food frequency questionnaires (FFQ) were analysed in detail and the self-reported dietary pattern assessed with our criteria. If it was discovered that a participant does not follow criteria of a reported diet, such participant was included into the correct group. There were two such cases—one self-reported vegan was discovered to be in fact a vegetarian and one self-reported vegetarian was found to be vegan. The required sample size to compare four groups of participants calculated a priori with G*Power 3.1.9.7 (Heinrich-Heine-Universität Düsseldorf, Germany), assuming an α level of 5% and β level of 20%, and a medium effect size (d = 0.4), was n = 76. A total of 130 participants met the criteria, and for the present study, 89 participants were selected to obtain four equal groups that were homogenous by age, sex, and BMI.

The participants payed one visit to the faculty. An on-line meeting was organized beforehand, where the participants were given the instructions on how to fill the dietary intake diary and other questionnaires and were asked to arrive to the measurements in the morning after fasting and restraining from physical exercise for at least 12 h.

### Anthropometric measurements and blood withdrawal

After arrival to the faculty, the participants were asked to rest for 10 min, then systolic and diastolic blood pressure and heart rate were measured on the left upper arm, in a seated position, with an automatic device (automatic blood pressure monitor SEM-1, Omron Healthcare Company, Singapore). Body mass was measured in light clothing and without shoes, using Tanita BC 418MA (Tanita Corporation, Arlington Heights, IL, USA). Body fat mass, fat free mass, total body water and phase angle were measured in a lying position using a bioelectric impedance analyser (BIA) Bodystat Quadscan 4000 (Bodystat Ltd., GB). Then, venous blood samples were collected in 5 mL serum vacuum blood collection tubes.

### Serum biomarkers on inflammation and intestinal barrier integrity

Blood samples were left on room temperature for 30 min to allow clot formation and were then centrifuged at 2000 rpm for 10 min. Serum aliquots were immediately frozen and stored at − 80 °C until further analysis. CRP levels were measured using a Cobas c111 analyser (Roche, Basel, Switzerland) and a corresponding reagent. Serum LBP, IL-6, TNF-α and zonulin levels were determined in duplicate on a microplate reader (Tecan, Mannedorf, Switzerland) using human ELISA kits (BioVendor, Brno, Czech Republic for LBP, IL-6 and TNF-α, MyBiosource Inc., San Diego, USA for zonulin). Assay sensitivity was 0.5 ng/ml for zonulin, 0.13 ng/ml for LBP, 0.32 pg/ml for IL-6, and 0.13 pg/ml for TNF-α. Assays inter-assay and intra-assay CVs were typically < 10%.

### Lifestyle and physical activity questionnaires

Detailed description of the distributed lifestyle questionnaire was published before^54^. In brief, the questionnaires consisted of demographical questions (age, sex, family status, education, socio-economic status), questions about family health history (diseases, allergies, use of medications and antibiotics), sleep and work schedule, perceived quality of life, and substance use (alcohol, smoking, psychoactive substances). The International Physical Activity Questionnaire (IPAQ) was used to determine physical activity. Based on the data on the duration and intensity of physical activity daily energy expenditure in metabolic equivalent of task (MET) was determined^55^.

### Dietary intake and dietary inflammatory index

Participants completed food diaries in which they recorded their dietary intake for 3 days, one day at the weekend and 2 days during the week. They were instructed to weigh food and drink before consumption and to weigh any leftovers. They also recorded food labels and recipes, and the intake of dietary supplements. The data from the food diaries was analysed using the Open Platform for Clinical Nutrition (OPEN), accessible at https://opkp.si/, which allows automatic conversion into intakes of energy and nutrients. Daily intakes of protein, carbohydrate, fibre, total fat, saturated fatty acids (SFA), monounsaturated fatty acids (MUFA), polyunsaturated fatty acids (PUFA), and ω-3/ω-6 ratio, were calculated as the sum of dietary and supplement intake. Participants also completed FFQ validated for Slovene population^56^.

### Statistical analysis

Statistical analysis was performed using IBM SPSS Statistics, version 26.0 (IBM Corp., Armonk, NY). The normality of data distribution was evaluated using the Shapiro–Wilk test. Continuous descriptive variables are expressed as means with standard deviations (SD) or median with interquartile range (IQR), and discrete variables as the frequency (%) of participants. The Chi-Squared test was used for categorical variables. Groups of participants with four dietary patterns and groups of participants in eight clusters were compared using one-way ANOVA or Kruskal–Wallis test, and Tukey or Bonferroni post-hoc test. Additionally, nonparametric ANCOVA was used, where adjustments were performed for age, sex, smoking status, education, waist circumference, physical activity and alcohol consumption. Spearman’s Correlation was used to investigate associations between dietary intakes, physical activity, phase angle with inflammatory and intestinal permeability biomarkers. *p*-values < 0.05 were considered statistically significant. Moreover, hierarchical cluster analysis according to CRP, IL-6, TNF-α, LBP, and zonulin was performed using MATLAB 2020A.

## Data Availability

The authors confirm that the data supporting the findings of this study are available within the article. The additional data are available on request from the corresponding author.
